# Is ischemic core volume a valid argument to withhold thrombectomy from ischemic stroke patients with major cerebral artery occlusions?

**DOI:** 10.1007/s00234-023-03218-6

**Published:** 2023-09-02

**Authors:** Rüdiger von Kummer

**Affiliations:** Institute of Diagnostic and Interventional Neuroradiology, Universitätsklinikum Dresden, Fetscherstr. 74, 01307 Dresden, Germany

Dear Editor,

This letter to the editor comments on the results of 3 recent randomized controlled trials (RCTs) on the efficacy and safety of thrombectomy in stroke patients with large ischemic cores [1]. This topic is of very high interest for the readers of *Neuroradiology*. The authors conclude that the trials’ data do not support thrombectomy in patients with an ischemic core exceeding 150 ml and recommend a case-by-case decision for patients with larger cores than 150 ml. They do, however, not discuss the reliability and accuracy of ischemic core assessment in these 3 RCTs [2–4] and do not suggest how to safely identify ≥ 150 ml of ischemic core.

The Recovery by Endovascular Salvage for Cerebral Ultra-Acute Embolism–Japan Large Ischemic Core Trial (RESCUE-Japan LIMIT) has used non-contrast CT in 28 of 203 study participants (14%) and DWI in 175 study participants (86%) to determine the Alberta Stroke Program Early Computed Tomographic Score (ASPECTS) of early ischemic changes being considered “infarctions” without prove [2]. The treating neurologist and no radiologist evaluated the ASPECTS. Reliability tests or diagnostic accuracy studies were not performed. Ischemic core volume inclusion criterion was an ASPECTS of 3 to 5. Follow-up imaging was performed; the results, however, were not reported. The predictive value of admission DWI-ASPECTS for brain tissue damage (infarction) would have been of high interest.

The ANGEL-ASPECT investigators included all patients with an ASPECTS of 3 to 5 on non-contrast CT and, in addition, patients with an ASPECTS < 3 and > 5 if the “infarct-core volume” was between 70 and 100 ml [3]. They used RAPID software to evaluate ischemic changes on CT perfusion (CTP) maps of 417 study participants (92%) and on DWI in 38 study participants (8%). They defined the brain tissue with a relative cerebral blood flow of less than 30% based on CTP imaging or an apparent diffusion coefficient (ADC) value of less than 620 × 10^−6^ mm^2^ per second as “core infarct.” The full-analysis population included 60 study participants with an ASPECTS < 3 (24%). Follow-up imaging was performed at 36 h with MRI or at 7 days with non-contrast CT. The “change from baseline in infarct-core volume” is presented in table 2 without dimension and thus hard to interpret. It remains unknown in how many patients a shrinkage of the initial “infarct-core volume” was observed.

The SELECT2 investigators like the ANGEL-ASPECT investigators included all patients with an ASPECTS of 3 to 5 on non-contrast CT and in addition patients with an estimated ischemic-core volume of ≥ 50 ml on CTP maps (defined as a relative cerebral blood flow of < 30% as determined with RAPID software) or a lesion with a volume of ≥ 50 ml with an ADC of less than 620 × 10^−6^ mm^2^ per second [4]. Based on these definitions, they included 20 patients with an ASEPCTS of 0 to 2 and 42 patients with an ASPECTS of 6 to 10. One hundred twenty-four patients had an ischemic-core volume of < 70 ml and 44 of ≥ 150 ml.

Despite these different approaches for including stroke patients with an ASPECTS of 3 to 5 and the additional inclusion of patients with an ASPECTS < 3 and a volume of ischemic core ≥ 150 ml, thrombectomy being performed within 24 h after stroke onset was beneficial and not associated with symptomatic intracranial hemorrhages in all 3 RCTs irrespectively whether a mismatch profile was present [4]. This puts in question the utility of a time-consuming volumetry of “ischemic core” and favors to offering thrombectomy to all patients with ischemic stroke due to major cerebral artery occlusion. Moreover, the ADC threshold of 620 × 10^−6^ mm^2^ per second for the delineation of ischemic cores is based on a study in 14 patients only that showed a sensitivity of 69% and a specificity of 78% for the identification of ischemic core brain tissue [5].

In the past, very many ischemic stroke patients remained untreated because of unwarranted restrictions. Intravenous thrombolytics (IVT) were not recommended if patients presented after 3 h, although no RCT had shown that late treatment is associated with an increased risk. ECASS 3 then showed the small benefit of IVT if applied between 3 and 4.5 h after stroke onset [6]. The meta-analysis of individual patient data in 6756 study participants of RCTs of alteplase versus placebo did not show an association between symptomatic intracranial hemorrhage risk and treatment delay [7].

An ASPECTS of 0 means that all middle cerebral artery subterritories are affected by ischemic changes but tell nothing about the territory supplied by the anterior and posterior cerebral artery that may be at risk if the internal carotid artery (ICA) is occluded which was the case in 36 to 47% in the 3 RCTs discussed here. The ASPECTS of ischemic brain tissue changes should be considered only in relation to the arterial occlusion site, the collateral blood flow, and the National Institute of Health Stroke score. Any cut-off threshold may mean that ischemic stroke patients with a chance to improve with thrombectomy will not be treated.
